# Priority effects and density promote coexistence between the facultative predator *Chrysomya rufifacies* and its competitor *Calliphora stygia*

**DOI:** 10.1007/s00442-022-05175-y

**Published:** 2022-05-03

**Authors:** Blake M. Dawson, James F. Wallman, Maldwyn J. Evans, Nathan J. Butterworth, Philip S. Barton

**Affiliations:** 1 Centre for Sustainable Ecosystem Solutions, School of Earth, Atmospheric and Life Sciences, University of Wollongong, Wollongong, NSW Australia; 2 Faculty of Science, University of Technology Sydney, Ultimo, NSW Australia; 3 Fenner School of Environment and Society, Australian National University, Canberra, ACT Australia; 4 Department of Ecosystem Studies, Graduate School of Agricultural and Life Sciences, The University of Tokyo, Tokyo, Japan; 5 School of Life Sciences, University of Technology Sydney, Ultimo, NSW Australia; 6 Future Regions Research Centre, Federation University Australia, Mount Helen, VIC Australia

**Keywords:** Carrion, Competition, Insect succession, Necrobiome, Temporal partitioning

## Abstract

Highly competitive ephemeral resources like carrion tend to support much greater diversity relative to longer-lived resources. The coexistence of diverse communities on short-lived carrion is a delicate balance, maintained by several processes including competition. Despite this balance, few studies have investigated the effect of competition on carrion, limiting our understanding of how competition drives coexistence. We investigated how priority effects and larval density influence coexistence between two blowfly species, the facultative predator *Chrysomya rufifacies* and its competitor *Calliphora stygia*, which occupy broadly similar niches but differ in their ecological strategies for exploiting carrion. We examined how adult oviposition, larval survival, developmental duration, and adult fitness were affected by the presence of differently aged heterospecific larval masses, and how these measures varied under three larval densities. We found *C. rufifacies* larval survival was lowest in conspecific masses with low larval densities. In heterospecific masses, survival increased, particularly at high larval density, with priority effects having minimal effect, suggesting a dependency on collective exodigestion. For *C. stygia*, we found survival to be constant across larval densities in a conspecific mass. In heterospecific masses, survival decreased drastically when *C. rufifacies* arrived first, regardless of larval density, suggesting *C. stygia* is temporally constrained to avoid competition with *C. rufifacies*. Neither species appeared to completely outcompete the other, as they were either constrained by density requirements (*C. rufifacies*) or priority effects (*C. stygia*). Our results provide new mechanistic insights into the ecological processes allowing for coexistence on a competitively intense, ephemeral resource such as carrion.

**Supplementary Information:**

The online version contains supplementary material available at 10.1007/s00442-022-05175-y.

## Introduction

Interspecific competition is a common biotic mechanism that shapes and drives species community structure (Connell 1983; Goldberg and Barton 1992). On ephemeral resources—those resources that are limited, patchy and unpredictable—interspecific competition can be particularly intense owing to the fact that the resource can only host a finite number of individuals (Atkinson and Shorrocks 1981; Kneidel 1985). For example, numerous *Drosophila* larvae compete for resources on decaying fruit bodies (Krijger et al. 2001), while biotic communities at other ephemeral resources such as dung and leaf litter also exhibit strong interspecific competition (Finn and Gittings 2003; Murrell and Juliano 2014). Carrion, the decomposing remains of dead animals, is an ideal ephemeral resource to examine interspecific competition, as it hosts a diverse range of eukaryotic and prokaryotic species, collectively known as the ‘necrobiome’ (Benbow et al. 2019). While numerous studies have examined interspecific competition at the community level, very few studies have examined interspecific competition at the species level to determine the exact mechanisms regulating coexistence on an ephemeral resource (Ito 2020).

Competition on carrion varies dynamically over time in response to the carrion resource drastically changing in size and quality during the decomposition process (Dawson et al. 2021b). Small-bodied vertebrate carrion (e.g. rabbit) likely experience more pronounced interspecific competition because resources are more limiting than larger carrion (Denno and Cothran 1976). However, large-bodied carrion (e.g., pigs) do still experience interspecific competition, as they are not immediately available to colonise and exploit in their entirety (Matuszewski and Mądra-Bielewicz 2021). Colonisation, even on large carcasses, occurs initially at key access points such as orifices or wounds (Archer and Elgar 2003), with competition by maggots for space and resources likely to be intense (Smith and Wall 1997). Larvae of Diptera species are one of the most abundant taxon groups on carrion and are responsible for the majority of carrion biomass consumption by insect fauna (Payne 1965; Archer 2004). Diptera larvae aggregate together forming large maggot masses, often consisting of hundreds to thousands of individuals, which can be composed of multiple heterospecific larvae (Fouche et al. 2018). Despite being in direct competition for resources in a heterospecific maggot mass, the benefits of increased growth and development from thermal dynamics and collective exodigestion may outweigh the detrimental effects of competition (Ives 1991; Rivers et al. 2011; Barton et al. 2021; Charabidze et al. 2021).

Not all carrion flies display this interspecific social behaviour and aggregate together. For example, ‘smooth’ maggot species of *Calliphora* and *Sarcophaga* avoid aggregating with ‘hairy’ maggots, such as *C. rufifacies* (Fuller 1934; Yang and Shiao 2012; Pimsler et al. 2019). This is because *C. rufifacies* larvae are facultative predators of other blowflies, with predation thought to occur mostly when resources are limiting and competition is high (Baumgartner 1993). Due to this predatory behaviour, the larvae of *C. rufifacies* may have a large competitive advantage over the larvae of other blowfly species, which have little defence against predation (Supp. Movie S1). *Chrysomya rufifacies* has the ability to shape the successional process and community composition because other species will be outcompeted or avoid ovipositing entirely (Wells and Greenberg 1992; Yang and Shiao 2012). This is of particular importance in forensic entomology, as *C. rufifacies* might also influence the development time of other species, thereby impacting post-mortem interval (PMI) estimates derived from larval development rates (Swiger et al. 2014; Carmo et al. 2018).

Despite *C. rufifacies* displaying strong competitive behaviours, other blowfly species have been able to colonise and co-exist on carrion using numerous physiological and behavioural adaptions (Arias‐Robledo et al*.* 2019). A key adaptation displayed by some blowfly species is their ability to locate and colonise carrion within minutes after death, enabling them to exploit the resource before other species such as *C. rufifacies* arrive (Frederickx et al. 2012; Evans et al. 2020). Species arriving earlier than *C. rufifacies* may have time for their larvae to develop and avoid predation by *C. rufifacies* if they can reach a developmental optimum. *Chrysomya rufifacies* will gain an additional resource to feed upon if arriving shortly after a heterospecific blowfly species (Brundage et al. 2014)*.* However, what remains unknown is how the precise timing of *C. rufifacies* arrival, and the impact of larval densities on these priority effects, influence *C. rufifacies* survival and predation rate (Carmo et al. 2018). Importantly, what degree of temporal advantage do other blowfly species need to survive on a resource and successfully co-exist with a facultative predator like *C. rufifiacies*? Answering such questions will help to explain the mechanism for co-occurrence among species on other ephemeral resources by revealing their strategies for dealing with competition.

To address the above questions, we conducted a series of manipulative laboratory experiments analysing the role of priority effects and larval density on competition between *C. rufifacies* and another blowfly species, *C. stygia*. *Calliphora stygia* shares an overlapping geographic distribution and arrives at carrion at similar times to *C. rufifacies*. Despite this, these species co-exist abundantly in nature, with the exact mechanisms allowing for coexistence entirely unknown. To unravel these mechanisms, we tested two hypotheses:

1. That adult *C. stygia* will reduce oviposition on a resource that has already been colonised by *C. rufifacies* larvae. In contrast, adult *C. rufifacies* will increase oviposition on a resource that has already been colonised by *C. stygia* larvae.
2. That *C. stygia* larval survival rate and adult fitness would decrease, while development time would increase if this species arrived on a resource that was already colonised by *C. rufifacies* larvae, particularly at higher larval densities where competition is predicted to be more intense. In contrast, *C. rufifacies* larval survival and adult fitness would increase, while development time would decrease if arriving at a resource after *C. stygia* larvae, regardless of larval density.

## Materials and methods

### Insect colonies and maintenance

To establish laboratory colonies, we purchased *C. stygia* pupae from a commercial supplier (Sheldon’s Bait). *Chrysomya rufifacies* adults were collected from wild populations around the University of Wollongong (UOW), Australia, and provided with kangaroo mince for oviposition. Once adults emerged from the pupae, they were transferred to a large plastic colony cage (300 mm × 500 mm × 250 mm) with a fly screen lid and provided with a constant supply of granulated raw sugar and water. A small amount of kangaroo mince (– 20 g) was provided to recently emerged adults to act as a protein meal, which females need for ovarian development (Cook 1991). To establish a new generation, we provided adults with – 50 g of kangaroo mince in a weigh boat, with cotton wool placed on top of the mince to replicate mammalian fur. Once oviposition occurred, the mince was removed from the colony cage and placed in a small plastic rearing container (130 mm × 190 mm × 70 mm) with the bottom of the container layered with wheaten chaff to act as a pupation substrate. Once hatched, the larvae were provided with a constant supply of kangaroo mince until pupation to ensure food was not limiting. Upon adult emergence, we placed the new generation into a clean large colony cage. All colonies were maintained in a temperature-controlled room at 24 ± 1 °C) with a 12:12-h light/dark cycle.

### Adult oviposition experiment

To examine adult oviposition preference, we provided adults with kangaroo mince that either had different ages of heterospecific larvae feeding on it (2- or 4-day-old larvae) or no larvae present on the mince (control) (Supp. Fig S1). To attain heterospecific larvae, adults from the stock laboratory colonies were placed in a small plastic rearing container with a weigh boat containing kangaroo mince and a layer of cotton wool on top of the mince. We then transplanted these heterospecific larvae onto fresh (less than 1 day old) kangaroo mince (50 ± 1 g) 30 min prior to the addition of adults to create the three different treatments (+ 2 days old, + 4 days old and control). For each replicate, 10 adult flies (5 males and 5 females) and 20 heterospecific larvae (except for the control, which had no larvae) were used, for a total of 12 replicates per treatment. We conducted the experiment twice, once using adult *C. rufifacies* laying on mince with different age *C. stygia* larvae present, and a second time with the roles reversed using adult *C. stygia* and *C. rufifacies* larvae. All adults were sourced from laboratory stock colonies with adults being at least 9 days old (to ensure they were sexually mature (Cook 1991)) and had not previously laid. We placed treatments in temperature cabinets for a period of 4 h set at 25 ± 0.5 °C and 50% ± 10% humidity. Only one treatment was in a cabinet at any one time to avoid chemical cues from other treatments influencing adult oviposition behaviour. After the 4-h period, we removed treatments from the cabinets and counted the number of eggs laid by manually separating them from the mince using a damp paint brush.

### Larval competition experiment

To examine the role of priority effects and larval density on competition, we placed larvae of *C. rufifacies* and *C. stygia* of different ages into mixed maggot masses on kangaroo mince (Supp Fig. S1). Adult flies from the stock laboratory culture were provided with kangaroo mince for oviposition. Once eggs had hatched, we provided larvae with a constant supply of mince to ensure food was not limiting. Once they had reached the desired age, we then removed the larvae and transplanted them onto new fresh (less than 1 day old) kangaroo mince (50 ± 1 g) in a plastic weigh boat. We used five heterospecific priority effect treatments, which consisted of: 0-day-old *C. rufifacies* + 0-day-old *C. stygia* (0R + 0S), 2-day-old *C. rufifacies* + 0-day-old *C. stygia* (2R + 0S), 4-day-old *C. rufifacies* + 0-day-old *C. stygia* (4R + 0S), 0-day-old *C. rufifacies* + 2-day-old *C. stygia* (0R + 2S) and 0-day-old *C. rufifacies* + 4-day-old *C. stygia* (0R + 4S). We placed the kangaroo mince with the heterospecific treatments in a small plastic rearing container, with the bottom of the container layered with chaff. Two conspecific larval treatments were also created consisting of only 0-day-old *Ch. rufifacies* or 0-day-old *C. stygia* larvae.

We conducted the larvae priority effect treatments under three larval densities: 25 of each species (50 total), 50 of each species (100 total) and 75 (150 total) of each species. To standardise density in the conspecific treatments, the larval density matched the total density of the priority effect treatments. For example, 50 larvae were used for the priority effect conspecific control to match the density of the 25 larvae of each species in that treatment. Within each larval density, we used six replicates for each priority effect treatment (6 × 0R + 0S within the’25 each’ larval density). A total of 12,600 larvae were used across the experiment (6300 per species). We placed treatments into temperature cabinets for a period of 4 weeks set at 25 ± 0.5 °C and 50% ± 10% humidity with a 12:12-h light/dark cycle. We visibly assessed treatments daily and time until adult eclosion was recorded. After the 4-week period, we removed treatments from the temperature cabinets and counted the total number of flies that reached the adult life stage. The dry weight of each adult fly was weighed using a Mettler Toledo ML204 Newclassic ml Analytical Balance. We converted the weight in grams (g) into milligrams (mg) by multiplying by 1000. We also recorded the sex of all individuals that survived to adulthood.

### Data analysis

To assess adult oviposition preference in the first experiment, we compared the number of eggs laid for each species between heterospecific larvae age treatments (no larvae, 2-day-old and 4-day-old larvae). We used two generalised linear models (GLMs), one for each species, with larval age treatment as a fixed, categorical predictor variable (3 levels) and the number of eggs laid as a continuous response variable. We analysed the two species separately because they differ in the number of eggs laid per clutch (Mackerras 1933). We assumed a Poisson error distribution and a log-link function for both GLMs, unless the data were over dispersed, in which case we assumed a negative binomial error distribution and a log-link function.

For the second experiment, to determine the effects of priority and larval density on species competition, we conducted a set of GLMs on multiple response variables. The three response variables included: number survived to adult life stage, time until adult eclosion (days) and adult fitness (mass (mg)). For each species, we conducted three separate GLMs, one for each of the three response variables, totalling six GLMs. For each GLM, the predictor variables were priority effect (6 levels) and larval density (3 levels) which were treated as fixed, categorical factors. The GLMs compared the priority effect treatments to a control within each larval density treatment. For the control, we used the 0 + 0 treatment group because this represented no priority effect in a heterospecific mass. In the same analysis, we also compared the conspecific treatment group with the control to determine the effect of adding heterospecifics. To control for density in the survival analyses, we halved the survival data for the conspecific treatments, because this treatment had double the number of individuals, enabling us to compare to the control (0 + 0) treatment.

Again, for the count of emerged adults (survival), we assumed Poisson or negative binomial error distributions. For the models using continuous time until adult eclosion (days) and adult fitness (mass) variables we assumed a Gaussian error distribution. We plotted the GLM estimates as effect sizes (estimate of the coefficient from the model) and interpreted their effects as significant if their 95% confidence intervals did not cross the zero-effect line (du Prel et al. 2009). We conducted all GLMs using R (3.6.0) (R Core Team 2019), and the package glmmTMB (Brooks et al. 2017). All plots were created using the ggplot2 package (Wickham 2016).

## Results

### Adult oviposition preference

**Fig. 1:**
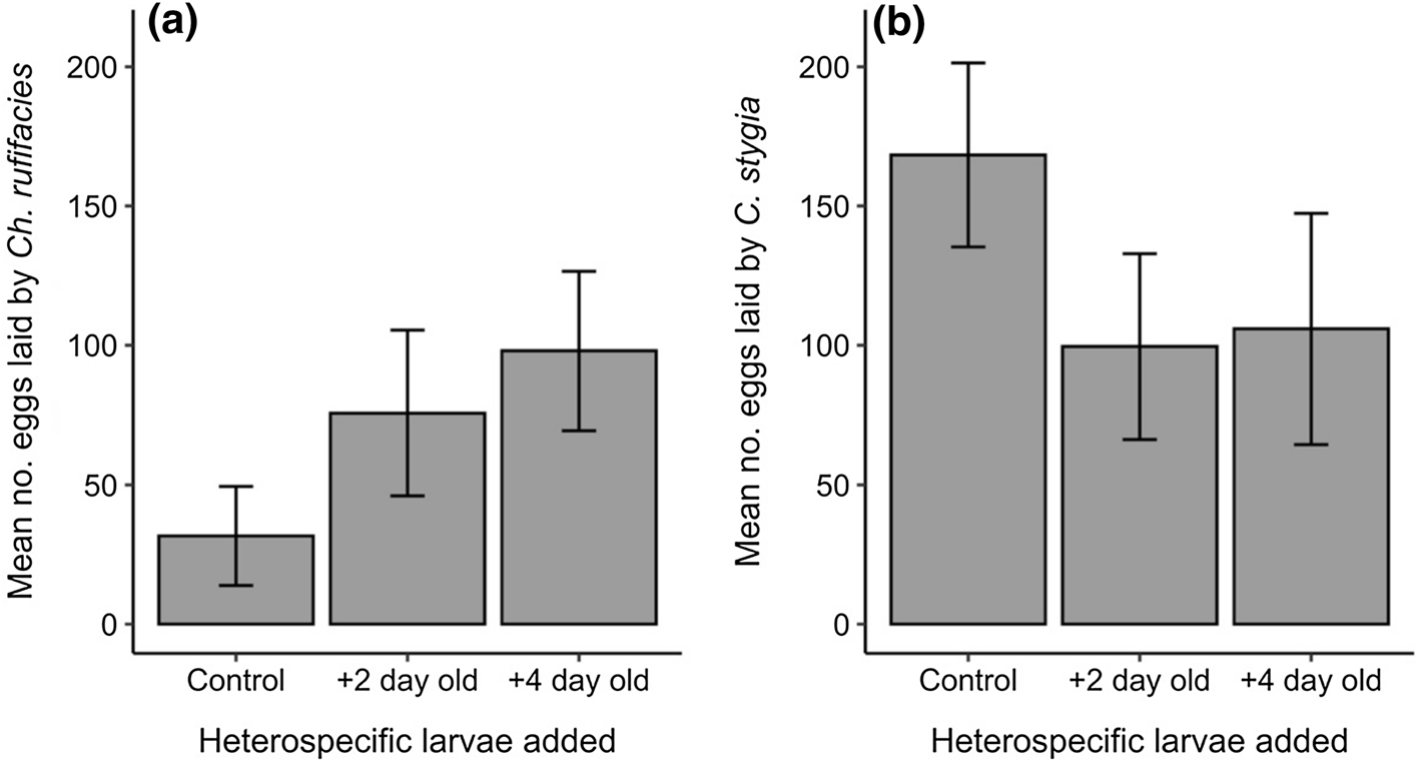
Bar plot of mean (± S.E.) number of eggs laid by adult **a** *Chrysomya rufifacies* and **b** *Calliphora stygia* on kangaroo mince with no larvae present (control), 2-day-old heterospecific larvae present (+ 2 day old) or 4-day-old heterospecific larvae present (+ 4 day old)

For *C. rufifacies*, the presence of both 2- and 4-day-old *C. stygia* larvae on a resource had no effect on the number of eggs laid by adults when compared to a resource with no *C. stygia* present (2 day GLM: intercept: 3.46, coefficient = 0.87, *t* = 1.44, *P* = 0.16; 4 day GLM: intercept: 3.46, coefficient = 1.13, *t* = 1.86, *P* = 0.07). There was a non-significant trend of increasing number of eggs laid when older heterospecific larvae were present on the resource (Fig. 1a). For *C. stygia*, we also found that the presence of both 2- and 4-day-old *Ch. rufifacies* larvae on a resource had a non-significant effect on number of eggs laid by adults when compared to a resource with no *Ch. rufifacies* (2 day GLM: intercept: 5.13, coefficient = − 0.53, *t* = − 1.17, *P* = 0.25; 4 day GLM: intercept: 5.13, coefficient = − 0.46, *t* = − 1.03, *P* = 0.31). Compared to *C. rufifacies* oviposition, the opposite trend was observed with *C. stygia*, as adults laid more eggs on a resource without *C. rufifacies* larvae present (Fig. 1b).

### Priority effects and larval density: survival to adulthood

**Fig. 2:**
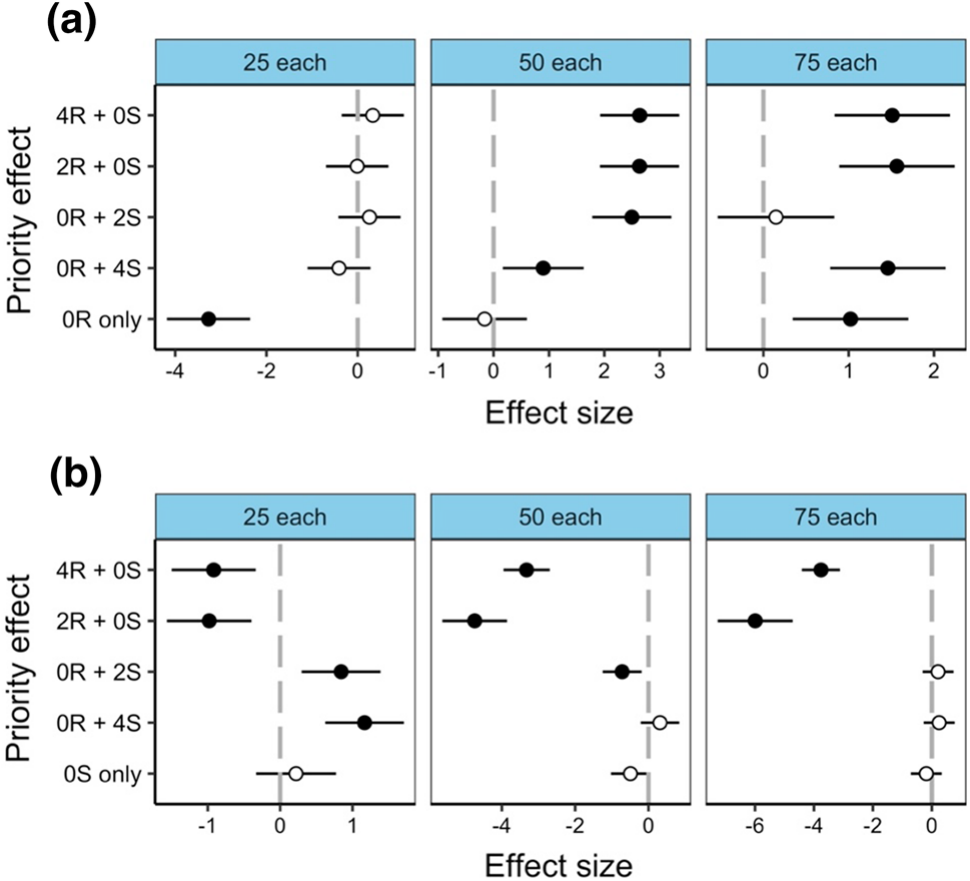
Effects of priority effect treatments on larval survival to adulthood relative to the control (0R + 0S; no priority effect in a heterospecific mass) within three different larval densities for **a** *Chrysomya rufifacies* and **b** *Calliphora stygia*. Priority effect treatments within heterospecific masses is on the y-axis, with numbers representing age of larvae (0, 2 or 4 days old) and letters representing species (R = *C. rufifacies* and S = *C. stygia*). Conspecific mass consisting of only one species is the bottom tick of the y-axis (0R only for *C. rufifacies* conspecific mass and 0S only for *C. stygia* conspecific mass). Significant effects (shown in bold) are denoted by 95% CIs that do not cross zero, which represents the control for priority effect (grey dotted line). Effect sizes are derived from GLMs

For the priority effect, survival was not significantly different from the control for *C. rufifacies* within the ‘25 each’ larval density (Fig. 2a). However, for the ‘50 each’ and ‘75 each’ larval densities, almost all priority effects had significantly higher survival than the control, except for 0R + 2S within the ‘75 each’ larval density, which was not significantly different from the control (Fig. 2a). Survival in a conspecific mass compared to the control (0R only) had a strong association with larval density. The conspecific mass had significantly lower survival in the ‘25 each’ larval density, was not significantly different in the ‘50 each’ larval density and significantly higher survival in the ‘75 each’ larval density (Fig. 2a). For *C. stygia,* we found the 4R + 0S and 2R + 0S priority effects had significantly lower survival than the control for all three larval densities (Fig. 2b). The other priority effects (0R + 2S and 0R + 4S) both had significantly higher survival than the control in the ‘25 each’ larval density but were not significantly different in the ‘75 each’ larval density. Conspecific survival was also not significantly different from the control in any of the three larval densities (Fig. 2b).

### Priority effects and larval density: development time until adult eclosion

**Fig. 3:**
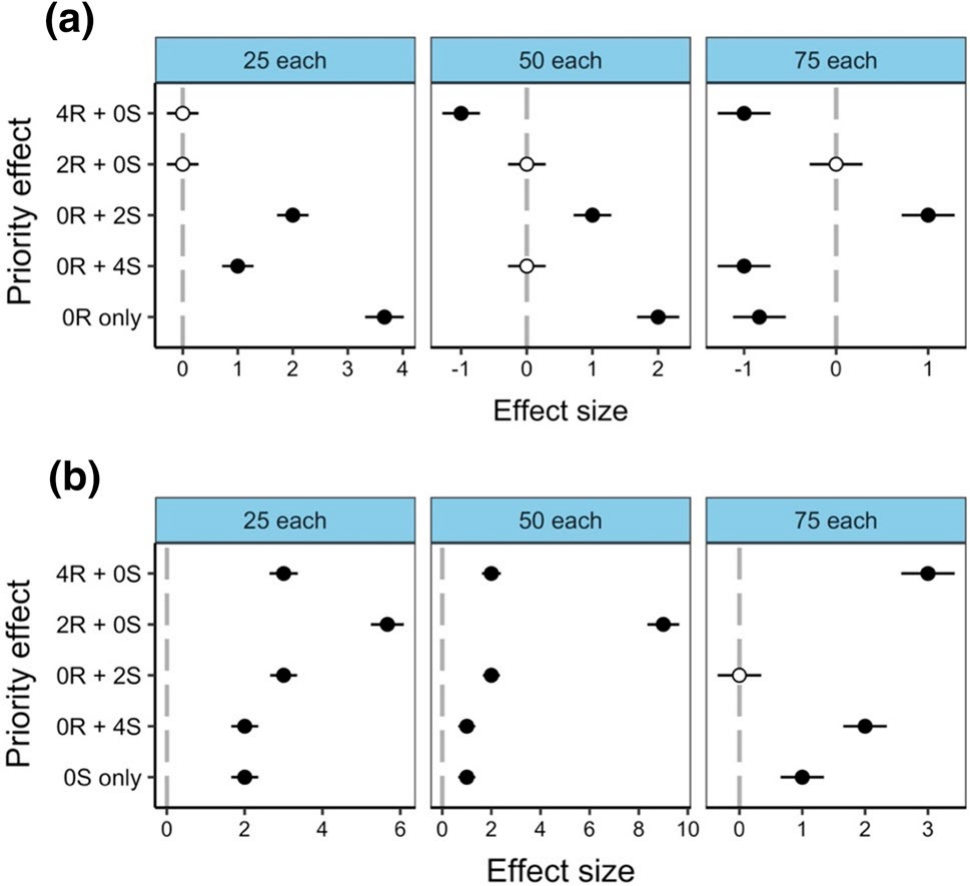
Effects of priority effect treatments on development time to adult eclosion relative to the control (0R + 0S; no priority effect in a heterospecific mass) within three different larval densities for **a** *Chrysomya rufifacies* and **b** *Calliphora stygia*. Priority effect treatments within heterospecific masses is on the y-axis, with numbers representing age of larvae (0, 2 or 4 days old) and letters representing species (R = *C. rufifacies* and S = *C. stygia*). Conspecific mass consisting of only one species is the bottom tick of the y-axis (0R only for *C. rufifacies* conspecific mass and 0S only for *C. stygia* conspecific mass). Significant effects (shown in bold) are denoted by 95% CIs that do not cross zero, which represents the control for priority effect (grey dotted line). Effect sizes are derived from GLMs

For *C. rufifacies* within the ‘25 each’ larval density, development time was significantly longer than the control for the 0R + 2S and 0R + 4S priority effects, but not significantly different for 4R + 0S and 2R + 0S (Fig. 3a). The ‘50 each’ and ‘75 each’ larval densities displayed similar results for *C. rufifacies*, with development time significantly shorter for 4R + 0S, and significantly longer for 0R + 2S. In the same larval densities, 2R + 0S displayed no significant difference in development time compared to the control, while 0R + 4S was not significantly different in the ‘50 each’ larval density but was significantly shorter in the ‘75 each’ larval density. The conspecific mass had significantly longer development times compared to the control for the ‘25 each’ and ‘50 each’ larval densities, but significantly shorter development time in the ‘75 each’ larval density (Fig. 3a). For *C. stygia* development time, almost all priority effects were significantly longer than the control, except for 0R + 2S in the ‘75 each’ larval density, which was found to be not significantly different from the control (Fig. 3b). The conspecific mass was also found to have significantly longer development time compared to the control for all larval densities.

### Priority effects and larval density: adult fitness

**Fig. 4:**
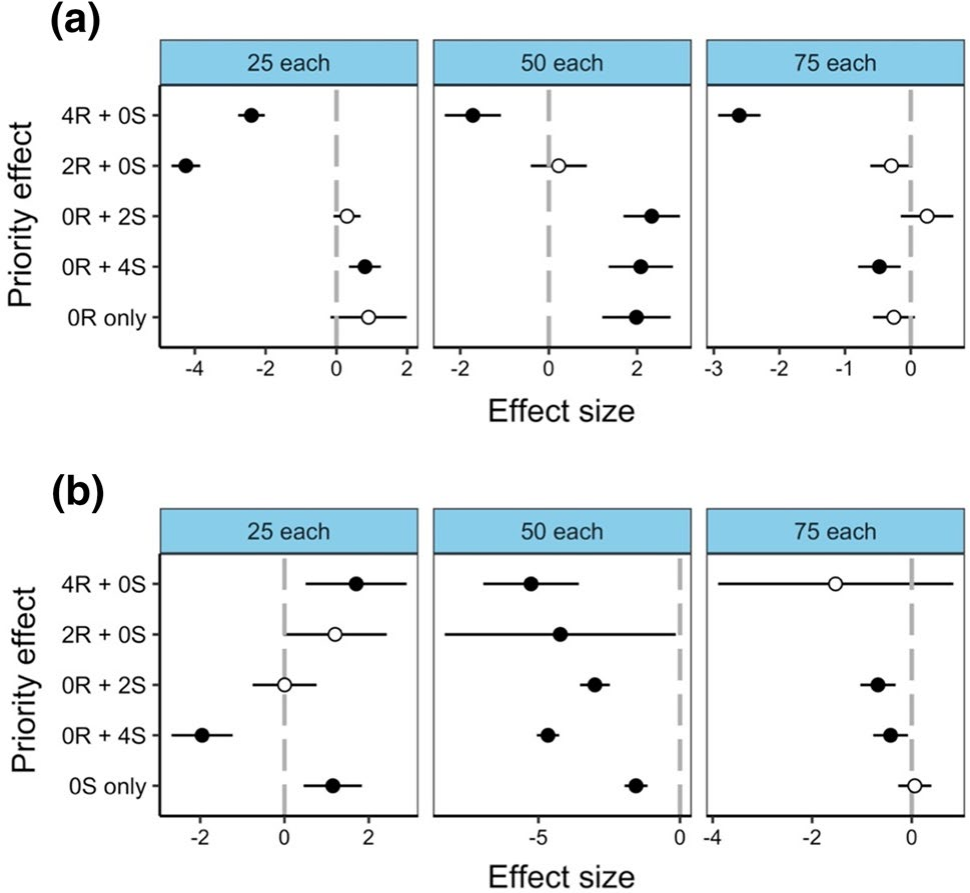
Effects of priority effect treatments on adult fitness (body mass (mg)) relative to the control (0R + 0S; no priority effect in a heterospecific mass) within three different larval densities for **a** *Chrysomya rufifacies* and **b** *Calliphora stygia*. Priority effect treatments within heterospecific masses is on the y-axis, with numbers representing age of larvae (0, 2 or 4 days old) and letters representing species (R = *C. rufifacies* and S = *C. stygia*). Conspecific mass consisting of only one species is the bottom tick of the y-axis (0R only for *C. rufifacies* conspecific mass and 0S only for *C. stygia* conspecific mass). Significant effects (shown in bold) are denoted by 95% CIs that do not cross zero, which represents the control for priority effect (grey dotted line). Effect sizes are derived from GLMs

For adult fitness (body mass), *C. rufifacies* had significantly lower fitness than the control for 4R + 0S and 2R + 0S priority effects in the ‘25 each’ larval density (Fig. 4a). In the same larval density, 0R + 2S was not significantly different from the control, while 0R + 4S had significantly higher fitness. The ‘50 each’ and ‘75 each’ larval densities shared similar results, with fitness found to be significantly lower than the control in 4R + 0S but not significantly different in 2R + 0S. The larval density did differ, however, in some priority effects, as 0R + 2S had significantly higher fitness in the ‘25 each’ larval density, but not significantly different in the ‘75 each’ larval density. By contrast, 0R + 4S had significantly higher fitness in the ‘50 each’ larval density but significantly lower fitness than the control in the ‘75 each’ larval density. The *C. rufifacies* conspecific mass was not significantly different from the control, except for the ‘50 each’ larval density, where it was significantly higher (Fig. 4a). For *C. stygia* fitness, almost all priority effects were found to be significantly lower than the control, particularly for the ‘50 each’ larval density. The exception to this was 2R + 0S and 0R + 2S in the ‘25 each’ larval density and 4R + 0S in the ‘75 each’ larval density which were all found to be not significantly different from the control. 4R + 0S in the ‘25 each’ larval density was the only priority effect found to have significantly higher fitness than the control. The conspecific mass when compared to the control displayed mixed results. It was found to be significantly higher in the ‘25 each’ larval density, significantly lower in the ‘50 each’ larval density and not significantly different in the ‘75 each’ larval density (Fig. 4b).

### Priority effects and larval density: overall comparison

**Fig. 5:**
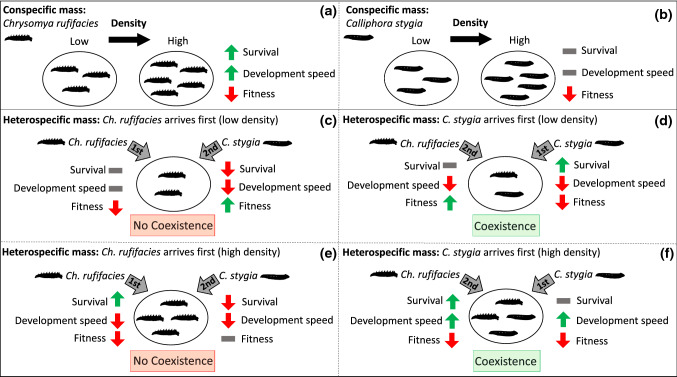
Conceptual diagram of larval density effects on conspecific masses of **a** *C. rufifacies* and **b** *C. stygia* larvae. The effect of different combinations of priority effects and larval density effects is also displayed for heterospecific masses: **c** *C. rufifacies* arriving first at low larval densities, **d** *C. stygia* arriving first at low larval densities, **e** *C. rufifacies* arriving first at high larval densities and **f** *C. stygia* arriving first at high larval densities. Coloured arrows represent changes relative to the 0 + 0 control for survival (survival to adulthood), development speed and fitness (body mass). Green up arrows = increased survival rate, faster development speed (quicker) and increased fitness, and vice versa for red down red arrows. Grey dashed lines represent no effect

When combining all the larval analyses, we can see *C. rufifacies* had low survival and slow development speed in low larval density conspecific masses, with survival and development speed increased as larval density increased (Fig. 5a). This trend was not observed in *C. stygia* conspecific masses as survival and development time did not change as larval density increased (Fig. 5b). In heterospecific masses where *C. rufifacies* arrived first, *C. stygia* survival was greatly reduced regardless of larval density (Fig. 5c, e). Survival rate of *C. stygia* only increased or was similar to the control (heterospecific mass with no priority effect) when *C. stygia* arrived first (Fig. 5d, f). *Chrysomya rufifacies* on the other hand only had increased survival in high densities, regardless of priority effect (Fig. 5e, f), with no effect observed in low density masses (Fig. 5c, d).

## Discussion

We conducted a series of laboratory experiments to determine how competition between *C. rufifacies* and *C. stygia* is influenced by priority effects and larval density. Our first hypothesis was not supported statistically, as we found that the presence of different aged heterospecific larvae on a resource had no significant effect on adult oviposition by either *C. rufifacies* or *C. stygia*. Our second hypothesis was supported, in part, with results showing *C. stygia* unable to survive when arriving after *C. rufifacies*, regardless of density. *Chrysomya rufifacies* survival increased when in high density heterospecific masses, regardless of priority effect. Together, our findings indicate that there are a complex array of outcomes resulting from competitive interactions between *C. rufifacies* and *C. stygia*. We discuss these findings below in relation to coexistence among species with different strategies for ephemeral resource exploitation.

### Adults of both species displayed no oviposition preference

Neither species displayed a strong oviposition preference between a resource that had heterospecific larvae and one without. These unexpected results contrast with previous research where *C. rufifacies* laid significantly more eggs on a resource with larvae of *Chrysomya megacephala* present than one without (Yang and Shiao 2012). Conversely, *C. megacephala* was found to have laid significantly more eggs on a resource without larvae of *C. rufifacies* (Yang and Shiao 2012). Several other studies have also demonstrated how egg laying behaviour of blowflies can be influenced by the presence of heterospecifics (Giao and Godoy 2007; Spindola et al. 2017). Although our results were non-significant, we did observe a similar trend in egg laying behaviour as seen in previous studies. However, if our results reflect what would happen under natural settings, then it may be the case that adult *C. stygia* are not be able to detect the presence of *C. rufifacies* larvae via visual or chemical cues on a resource as effectively as other species (Brundage et al. 2017). Similarly, adult *C. rufifacies* may not display an obvious preference for a resource with or without *C. stygia,* as the fitness benefits from the heterospecific treatments may not differ considerably from a resource without heterospecific larvae present. *Chrysomya rufifacies* larvae may only become predatory when the carrion resource is limited. Therefore, if the resource is plentiful, adults may display no oviposition preference for the presence of heterospecific prey (Gomes et al. 2007; Pimsler et al. 2019). Alternatively, the number of heterospecific larvae on the resource in our experiments might not have been large enough to elicit a significant response from either species, or the 30 min interval for feeding was insufficient to produce heterospecific volatiles detectable by adults. It is possible that further replication may have increased the trends we observed and towards a significant result—so modification of oviposition behaviour should not be ruled out as an adaptation in either species.

### *Chrysomya rufifacies* is dependent on larval mass size

We observed that *C. rufifacies* survival decreased in conspecific larval masses with low density. The enhanced survival of *C. rufifacies* in high densities may be explained by the fact that this species, like many other blowflies, likely feeds on carrion resources via exodigestion mechanisms—which involve adult and larval flies excreting enzymes to breakdown food particles to a liquid state before ingestion (Scanvion et al. 2018). Exodigestion is made more efficient when larval mass size is increased and enzyme production is greater due to collective gregarious behaviour (Scanvion et al. 2018; Charabidze et al. 2021). For example, *Lucilia sericata* larvae had high mortality in low density masses on a fresh resource (Scanvion et al. 2018). However, when the same resource was altered to be more digestible, mortality rate decreased in low density masses, while high density masses had low mortality rates on either resource (Scanvion et al. 2018). This suggests that *L. sericata* requires a minimal larval density for effective exodigestion on a fresh resource. On fresh resources, *C. rufifacies* is likely under similar larval mass density requirements, as the resource has not yet been broken down by bacteria or other species secreting enzymes.

This requirement of collective gregarious behaviour may also explain why *C. rufifacies* survival rate increased in heterospecific larval masses at high larval densities regardless of the priority effect treatment. The resource may be more effectively broken down by the heterospecific larvae, enabling *C. rufifacies* to successfully feed on the fresh resource and thereby enhance its survival and reduce development time (Komo et al. 2019; Charabidze et al. 2021). Alternatively, *C. rufifacies* may use the heterospecific mass as an additional food source. However, because predatory behaviour is only exhibited by second and third instars, predation is likely only occurring in the age treatments where *C. rufifacies* was older (Baumgartner 1993). The requirements of minimum mass size for exodigestion or presence of heterospecific larvae are likely reasons why *Ch. rufifacies* generally acts as a secondary coloniser—laying larvae later in the decomposition process, even though adult flies arrive at carrion relatively early (Dawson et al. 2021a). It is unknown what morphological or physiological factors limit *C. rufifacies* exodigestion capabilities on fresh carrion, but these might relate to the larval mouthparts or the type of enzymes produced (Shiao and Yeh, 2008). It is clear that coexistence with a heterospecific is beneficial for *C. rufifacies* survival and life history traits, despite the detrimental effects of interspecific competition.

Fitness of *C. rufifacies* generally decreased when in heterospecific masses with younger *C. stygia* regardless of larval density. A reduction in fitness is likely due to the increased survival rate in the same priority effect treatments. *Chrysomya rufifacies* survival was increased in these conditions, resulting in more conspecifics on the resource and subsequently higher levels of interspecific competition, leading to reduced fitness of individuals (Peters and Barbosa 1977). In nature, there are likely trade off decisions that adults must make. For example, they might either lay in high densities where individuals are more likely to survive but have reduced fitness, or risk laying in low densities where survival is reduced, but those that do survive will be more fit (Raitanen et al. 2014). This type of decision making is crucial on ephemeral resources due to the limiting nature of the resource. With survival so low in conspecific masses, *C. rufifacies* has likely evolved to favour ovipositing in high density masses due to their potential reliance on collective exodigestion. The facultative predatory behaviour of *C. rufifacies* may be an additional adaption to allow them to cope better in high density larval masses and thereby reduce reliance on the carrion resource directly (Polis 1981).

### *Calliphora stygia* is dependent on priority effects

We found *C. stygia* larval survival to be substantially reduced when arriving after *C. rufifacies*. Survival of *C. stygia* and coexistence with *C. rufifacies* on a resource is only likely when they arrive at the same time or earlier than *C. rufifacies*. In these situations, *C. stygia* can feed and grow before *C. rufifacies* is able to reach a developmental stage at which they can display predatory behaviour (Brundage et al. 2014). Therefore, *C. stygia* survival on carrion can be mediated by the species arriving before *C. rufifacies*, thereby displaying temporal partitioning as a response to interspecific competition (Brundage et al. 2014). Temporal partitioning has also been shown to promote coexistence in other carrion species such as scavenging vertebrates (Olea et al. 2022), and on other ephemeral resources, such as wasp larvae on developing figs (Ranganathan et al. 2010). These examples of temporal partitioning highlights its importance in maintaining biodiversity on competitively intense ephemeral resources generally.

*Calliphora stygia* also had superior survival rates than *C. rufifacies* in low larval densities. *Calliphora stygia* likely has more effective feeding capabilities on a fresh resource potentially due to mouthpart morphology or type of enzymes produced, and does not have a lower threshold of larval mass size (Goodbrod and Goff 1990; Scanvion et al. 2018). This adaption to feeding on fresh remains that are less digestible to *C. rufifacies* has also likely led to the emergence of temporal partitioning behaviour on carrion (Barton et al. 2019; Benbow et al. 2019). *Calliphora stygia*, along with other Diptera and bacterial species alter the biochemistry of carrion, making the remains better suited to species like *C. rufifacies* (Tomberlin et al. 2017). This form of resource manipulation follows the facilitation model of succession which has not been well supported in carrion systems (Michaud and Moreau 2017), but has in other ephemeral resources such as decaying wood (Vindstad et al. 2020). More research is needed on fine-scale species interactions on carrion to determine the mechanisms shaping successional patterns and biodiversity.

The presence of younger and older heterospecifics also increased development time of *C. stygia*. Slower development rates are likely due to increased pressure from interspecific competition or spending a greater amount of time exhibiting predator avoidance behaviour, thereby resulting in less optimal resource and nutritional uptake (Wells and Kurahashi 1997). Altered development rates in heterospecific masses are an important consideration if larvae are used in forensic entomology for PMI estimates. Ideally, when estimating a PMI, forensic entomologists should not only consider the size of larval masses, but also whether they consist of one or more species.

### Implications and conclusions

Our results suggest that neither fly species can completely outcompete and dominate the other, as they are constrained by density requirements (*C. rufifacies*) or priority effects (*C. stygia*). The two blowfly species use different morphological and behavioural adaptions to survive on carrion, particularly *C. rufifacies*, which has evolved facultative predatory behaviour, possibly in response to its reliance on high larval densities for survival. The evolutionary processes underpinning the transition from obligate necrophage to facultative predator in this species requires more investigation. Conversely, *C. stygia* is reliant on priority effects and temporal partitioning for survival as a consequence of the competitively superior *C. rufifacies*. Broadly, our study shows how priority effects enable coexistence on carrion, and likely other ephemeral resources where competition is intense. The successional patterns observed on carrion and other resources, like dung or leaf packs, is generally the outcome of intense competition and subsequent temporal partitioning, with each species employing a range of morphological and behavioural adaptions to maximise survival potential. Further examination of these fine-scale species interactions and the outcomes of competition will enable researchers to determine the exact drivers of succession and coexistence on ephemeral resources.

## Supplementary Information

Below is the link to the electronic supplementary material.

Supplementary file1 (DOCX 64 KB)

Supplementary file2 (DOCX 13 KB)

## Data Availability

The datasets used and/or analysed during the current study are available from the corresponding author on reasonable request.

## Code availability

The code used during the current study are available from the corresponding author on reasonable request.
