# Supplementary material for: Priority effects and density promote coexistence between the facultative predator Chrysomya rufifacies and its competitor Calliphora stygia
Source: Oecologia. 2022 May 3;199(1):181–91. doi: 10.1007/s00442-022-05175-y (PMC9119899; doi:10.1007/s00442-022-05175-y)
Supplement: Supplementary file 1 — Supplementary file1 (DOCX 64 KB) [file 442_2022_5175_MOESM1_ESM.docx]

**Supplementary material**

**Figure S1** Treatments and experimental design for experiment one (adult ovipositional preference) and experiment two (priority effects and larval density).
