# Supplementary material for: Priority effects and density promote coexistence between the facultative predator Chrysomya rufifacies and its competitor Calliphora stygia
Source: Oecologia. 2022 May 3;199(1):181–91. doi: 10.1007/s00442-022-05175-y (PMC9119899; doi:10.1007/s00442-022-05175-y)
Supplement: Supplementary file 2 — Supplementary file2 (DOCX 13 KB) [file 442_2022_5175_MOESM2_ESM.docx]

**Supplementary material**

<https://www.youtube.com/watch?v=xpvqeRy_oTI>

**Movie S1** Video displaying a mass of *Chrysomya rufifacies* preying upon a single *Calliphora stygia*.
